# Systematic perturbation of an artificial neural network: *A step towards quantifying causal contributions in the brain*

**DOI:** 10.1371/journal.pcbi.1010250

**Published:** 2022-06-17

**Authors:** Kayson Fakhar, Claus C. Hilgetag

**Affiliations:** 1 Institute of Computational Neuroscience, University Medical Center Eppendorf, Hamburg University, Hamburg, Germany; 2 Department of Health Sciences, Boston University, Boston, Massachusetts, United States of America; Ghent University, BELGIUM

## Abstract

Lesion inference analysis is a fundamental approach for characterizing the causal contributions of neural elements to brain function. This approach has gained new prominence through the arrival of modern perturbation techniques with unprecedented levels of spatiotemporal precision. While inferences drawn from brain perturbations are conceptually powerful, they face methodological difficulties. Particularly, they are challenged to disentangle the true causal contributions of the involved elements, since often functions arise from coalitions of distributed, interacting elements, and localized perturbations have unknown global consequences. To elucidate these limitations, we systematically and exhaustively lesioned a small artificial neural network (ANN) playing a classic arcade game. We determined the functional contributions of all nodes and links, contrasting results from sequential single-element perturbations with simultaneous perturbations of multiple elements. We found that lesioning individual elements, one at a time, produced biased results. By contrast, multi-site lesion analysis captured crucial details that were missed by single-site lesions. We conclude that even small and seemingly simple ANNs show surprising complexity that needs to be addressed by multi-lesioning for a coherent causal characterization.

## Author summary

The motto *“No causation without manipulation”* is canonical to scientific endeavors. In particular, neuroscience seeks to identify which brain elements are causally involved in cognition and behavior, by perturbing them. However, due to multi-dimensional interactions among the elements, this goal has remained challenging. Here, we used an Artificial Neural Network as a ground-truth model to compare the inferential capacities of two principal approaches, lesioning a system one element at a time versus sampling from the set of all possible combinations of lesions. We show that lesioning one element at a time provides misleading results. Hence, we argue for employing exhaustive perturbation regimes. We further advocate using simulation experiments and ground-truth models to verify the assumptions and limitations of current approaches for brain mapping by perturbation.

## Introduction

One of the most challenging goals of neuroscience is to identify neural elements–brain regions, populations, neuronal circuits, and large-scale networks–that are responsible for cognition and behavior [1]. During the past two decades, brain mapping has flourished with the help of neuroimaging techniques that are employed to statistically associate neural elements and brain functions. Arguably, however, the historical method of mapping brain function, by studying brain lesions and the resulting functional deficits (cf. [2]), still holds an authoritative role in establishing causation, since it indicates the necessity of elements for a given function [3,4]. In contrast to correlational approaches that quantify the statistical association between system elements and functions [5], perturbing and manipulating the system results in changes that can be used to pinpoint actual causes, since the manipulation is assumed to differentiate contributing causes from covarying confounds [6,7]. Historical cases of lesion inference after brain damage in patients [8] as well as modern cutting-edge experimental approaches employing opto- and chemogenetics [9,10] have mainly followed a “Single-element Perturbation Analysis (SPA)” framework. In this framework, *one neural element is perturbed at a time*. For instance, a classical lesion study considers a causal contribution for a brain element *i* with respect to a behavior *X*, if a lesion in *i* results in a deficit in *X*. By contrast, a more elaborate “double dissociation” study is assumed to further disentangle causal contributions of pairs of regions. If lesioning region *i* results a behavioral deficit in *X* and not in behavior *Y*, while ablation of a different region *j* causes a deficit in *Y* and not *X*, then *i* is considered to be a key player in producing *X* and *j* in producing *Y*. Moreover, the contributions of *i* and *j* are considered to be independent. Importantly, the perturbations are also independent and are sequentially performed, therefore, this approach is a subset of the SPA framework.

### Shortcomings of the Single-element perturbation analysis framework

Although “no causation without manipulation” [11] is indisputably true and points towards the eminence of perturbation approaches for identifying causal relationships in the brain, the practical and methodological difficulties of perturbation inferences are still underexplored [12,13]. Namely, previous studies have shown that localized perturbations result in network-wide effects even in the absence of direct connections from the perturbed site to spatially distant sites, a phenomenon known as ‘diaschisis’ [14–16]. Diaschisis demonstrates that neural elements are not independent, but are in fact linked functionally or structurally [13]. Therefore, perturbation approaches may suffer from covarying confounds, similar to correlational approaches [3,13]. However, there prevails an intuitive assumption that a large or even an exhaustive number of sequential perturbations can overcome these confounds and provide sufficient insight into the causal elements of a system.

The practical merits of the SPA framework were recently put to the test in an influential study by Jonas and Kording [17]. They performed an exhaustive lesioning of every transistor in a simulated microprocessor, to investigate if the approach provides a meaningful causal picture of the system. They defined the microprocessor’s behavior as its ability to boot different games, which allowed them to differentially map disrupted behaviors, that is, a failure in booting games, to the deactivated transistors. Moreover, their choice of a microprocessor as a ground-truth model provided them with confound-free access to virtually every computational unit [17]. As expected, they found subsets of transistors that, if perturbed, would disrupt specific behaviors. However, they declared the results “grossly misleading”, since “The transistors are not specific to any one behavior (…) but rather implement simple functions” [17]. Thus, Jonas and Kording [17] demonstrated that, even by perturbing *all* elements of a system, *one at a time*, we are still far from a coherent understanding of the characteristic causal functional contributions and interactions of the elements of a system.

Further inferential limitations of the SPA framework were discovered after *simultaneous* perturbations of multiple brain regions. Paradoxical lesion effects and, in particular, the “Sprague effect” are intriguing phenomena in this context [18,19]. The Sprague effect describes a setting in which disruptions in behavior caused by a first lesion revert towards normal behavioral state after a second lesion [19,20]. In other words, lesioning region *i* disrupts the given behavior *X*, providing apparently compelling evidence for its necessity for the behavior. Surprisingly, however, a subsequent lesion to another region *j*, while *i* is still perturbed, partly or completely restores the behavior *X*, showing the redundancy or degeneracy of the contribution of *i*. In this case, SPA can misattribute causal contributions. This includes double dissociation studies since they still draw conclusions from lesioning one element at a time [14].

### Summary of the current study

Paradoxical lesion effects, where the lesioning of two (or potentially more) elements may lead to unpredictable outcomes, and the practical findings of Jonas and Kording demonstrate that a SPA of every element does not provide a reliable causal picture, even if performed exhaustively. Therefore, a systematic approach that covers *all possible combinations of lesions* is needed as the basis for an objective multivariate evaluation of the system by perturbation.

Consequently, in this study, we used the approach of “Multi-perturbation Shapley value Analysis” (MSA) that, in contrast to SPA, derives causal contributions of elements from all combinations of multi-element lesions [21,22]. MSA is based on the Shapley value, a game-theoretical metric used for fair distribution of the outcome of a coalition, among members of it [23]. In the context of neuroscience, members are arbitrarily defined neural elements of a system that then will be ranked according to their contributions to a quantified behavior or cognitive function [24,25] that is, the coalition’s outcome.

We used an ANN as our ground-truth model and systematically and exhaustively perturbed its components. Specifically, we targeted all neurons and all connections, either one element at a time via SPA, or combinations of elements by MSA. To train the network, we specifically used an evolutionary algorithm focused on the network’s topology to avoid handcrafting and potentially biasing its organization.

We found that not every perturbation necessarily revealed causation. Although data from both lesioning regimes showed similarities, SPA missed some of the key contributing elements and miss-attributed their causal ranks. Therefore, SPA provided biased contributions for individual elements, while the MSA captured these nuances more accurately. To further quantify the complex interaction of elements within the system, we used an extension of MSA, here called Pairwise Causal Interaction Analysis (PCIA) [21,22], and found a handful of element pairs in which lesioning one unit while the other was perturbed restored the disrupted behavior.

Finally, we investigated the intrinsic mechanisms of the network to identify why MSA ranked the units in the given way and what these units do that SPA was insensitive to. We found that the most critical functional contributions originated from connections rather than nodes and in particular from connections underlying mutual inhibition and self-inhibition of two nodes. We discuss the findings, the limitations of the current approach, and potential questions for future research.

## Results

Our *in-silico* experimental setup was the ATARI arcade game Space Invaders [26], in which an agent, located at the bottom of the environment, needs to defend itself by using laser canons from aliens descending from the upper part of the screen. The main objectives of the game are to stay alive by avoiding alien laser shots and scoring as many points as possible by eliminating aliens. On average, a human subject obtains a score of 1652, and an algorithm that randomly selects actions reaches a score of 148 [27]. Other classic algorithms, such as an earlier implementation of a Deep Q-learning Network (DQN), State–Action–Reward–State–Action (SARSA), and a refined DQN, reach scores of 581, 271, and 1976, respectively [27,28].

Instead of training deep networks using backpropagation in a predefined architecture, we evolved a compact network using a Neural Architecture Search (NAS) algorithm called Neuro Evolution of Augmenting Topologies (NEAT) [29]. Briefly, NEAT uses evolutionary principles, such as cross-over of genes (network topologies), speciation (preserving novelty), and incremental complexification to find the “fittest” topology. This means that the network’s architecture and connectivity are not handcrafted, nor does the algorithm solely optimize connection weights. Instead, the fittest network is evolved with respect to the environmental constraints, in this case to achieve the highest score by adjusting its topology according to a set of given limitations, for instance, low probability of adding connections versus higher probability of removing them after each evolutionary step (see section *Evolutionary Optimization*).

**Fig 1:**
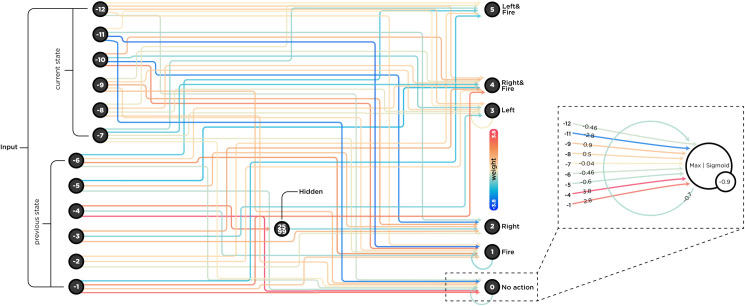
The complete wiring diagram of the evolved ANN. The network received a compressed version of the game-state as a vector of 12 features, six features per frame at each time point. It then chose an action from six available actions (output nodes). Due to its importance, which was revealed later in the analysis, we plotted node 0 separately with additional information on the right part of the figure. The aggregation function for this node is *max*, the activation function is a *sigmoid* function, and the *bias* is -0.9. Note that these settings are different for each node (see section *Evolutionary optimization*).

In addition to these sets of hyperparameters and to further enforce a compact architecture, we compressed the game frames using a deep auto-encoder and fed two feature vectors into our network, amounting to a total of 12 features at each time point, see neurons labeled with negative numbers in Fig 1. We fed in two frames instead of one due to the non-Markovian structure of the game in which knowing only the current position of laser beams does not provide enough information about the beams’ directions.

On average, our evolved network obtained a score of 337 that is significantly higher than that of a random agent with a score of 148 (Mann-Whitney U statistics; MWUs = 39542, p-value <0.001, S1 Fig). In order to ensure that the score was not merely higher because of innate topological privileges, we further compared the performance with the performance of two control networks. In one, we kept the network as it was and fed it with noise instead of features. In the other, we shuffled the connection weights while the network was receiving game-states. Both control networks obtained substantially lower scores, from 175 (MWUs = 50919, p-value <0.001) to 129 (MWUs = 31157, p-value <0.001), respectively. Altogether, these results show that our compact network did learn the task to some degree and could reach a good-enough score (S1 Fig) that formed the basis of the subsequent perturbation analyses.

### Perturbing all elements, one at a time

**Fig 2:**
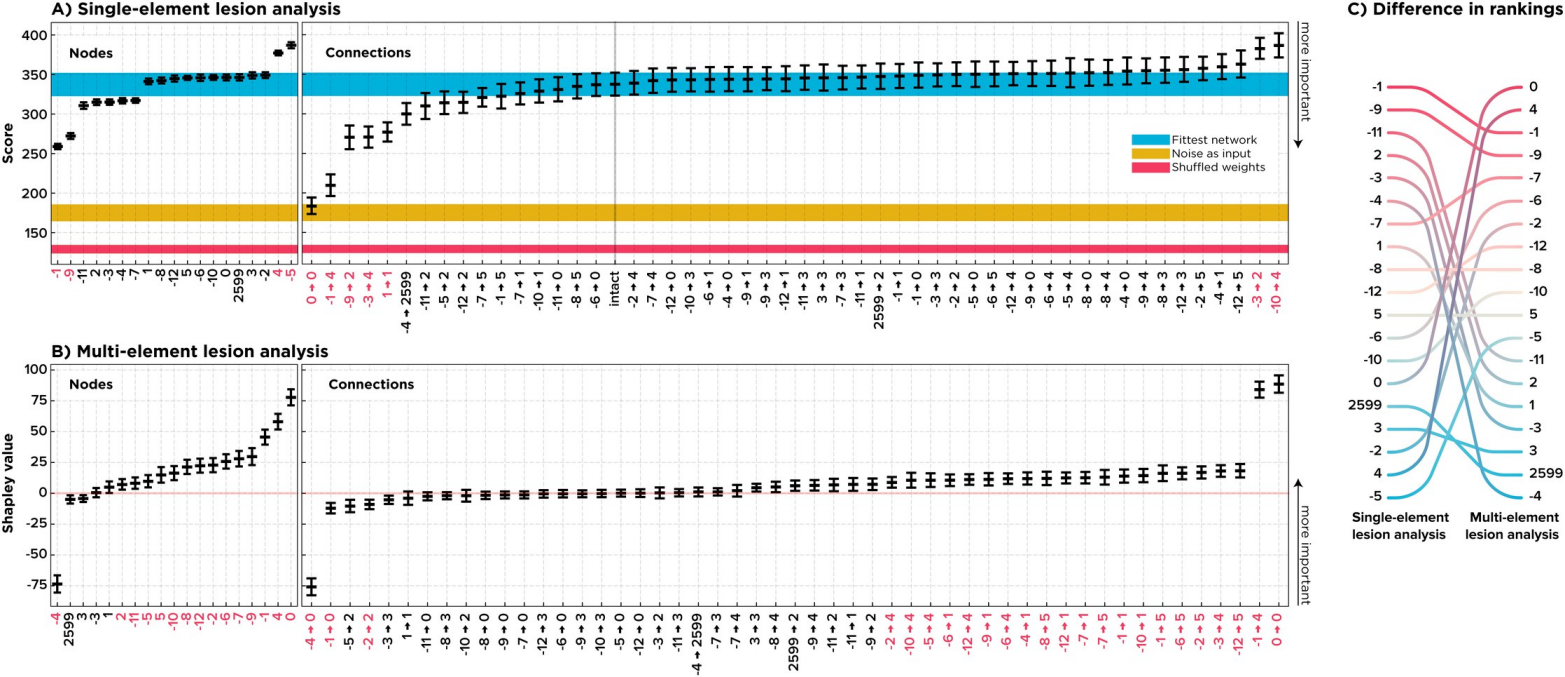
Single-element Perturbation Analysis versus multi-perturbation Shapley value Analysis of the ANN. This figure shows the result and the rank difference derived from a SPA (A; 512 samples per element) versus an MSA (B; 1,000 samples per element). On the left side the nodes, and in the middle the connections, are sorted according to their inferred average contributions. For SPA, the lowest value means the most influential while the other way around applies to Shapley values, with the highest value means the most critical. Error bars indicate %95 Confidence Interval (CI; bootstrapped 10,000 times). The blue, yellow, and red strips show the %95 CI of the labeled control networks. Red labels on the x-axes show significant elements (alpha inflation is corrected using Bonferroni correction, see *Statistical Inference* in *Materials and Methods*). On the right-hand side, the node rankings are compared.

After evolving the network, we intervened to establish if the perturbation of elements could reveal their causal importance for the behavior. We first silenced neurons one at a time and re-ran the simulation with the lesioned networks. Conventionally, we searched for neurons, which, when lesioned, resulted in a considerably deteriorated performance, indicating their “necessity” for the network behavior. As (Fig 2A) shows, lesioning either of two input neurons nodes -1 or -9, had such a disruptive impact, while individually perturbing most other neurons had a negligible effect on the performance. Interestingly, lesions of two neurons, nodes 4 and -5, improved the performance, suggesting their hindering role during normal functioning.

To account for the consequences of lesioning network connections rather than nodes, we performed the same lesioning scheme on all connections. Specifically, wanted to test if severing individual connections of neurons, instead of silencing a whole node with all its connections, could further localize functional contributions in our ANN. For example, should neurons -1 and -9 be considered as essential elements for the behavior of the ANN, or are there connections of these neurons such that the neurons only appear to be critical in the sense that lesioning them perturbs those connections as well? In this context, we expected to see either one of two potential outcomes. Either no specific connections would appear to be causally crucial, showing that the selected neurons were the actual units of causation, or a major disruption in behavior might follow lesions to the outgoing connections of neurons -1 and -9. In this case, the nodes would just appear to be critical because in fact the outgoing connections were critical.

Surprisingly, a loop from neuron 0 to itself (self-loop) appeared to be the most critical element (Fig 2A). This observation implies that, although SPA of all elements resulted in some degree of coherence, by first capturing neurons -1 and -9 as major players and then tracking their importance to connections (-1 → 4) and (-9 → 2), another key player was missed. Firstly, lesioning single connections did impact the performance considerably. Secondly, the most critical connection was not a connection from or to the most important neurons, but a self-loop of a neuron that itself had a near-zero causal contribution.

To summarize, results from the SPA of each neuron indicated that ablation of neuron 0 had little impact on the performance, while removing the self-loop (0 → 0) disrupted the behavior most strongly. Note that, throughout the lesioning experiments, there was no plasticity considered, the connection weights were fixed, and network’s architecture determined its behavior. Therefore, the fact that lesioning connection (0 → 0) alone caused the greatest damage, while lesioning neuron 0 with all its 11 connections including (0 → 0) did not produce any behavioral impairment, shows that higher-order perturbations are required for a more coherent characterization of the system. In the next section we describe how MSA infers causal contributions from such higher-order perturbations.

### Multi-perturbation shapley value analysis of all elements

Next, we adopted a multi-element lesioning approach, MSA, to systematically perturb combinations of all neurons and all connections. MSA is based on a game-theoretical concept called the Shapley value (γ). Shapley values account for the *“worth”* of elements in a system in terms of an element’s contribution to the overall system performance. The value is deduced from the element’s contribution to all possible combinations of groupings in the system [21–23]. To build an intuition of the problem, consider a group of people sharing the energy bill of their common office. The straightforward way to do so is to divide the cost equally, however, this is not necessarily the *fair* solution since they may not equally consume energy. A fair, and thus, stable solution should allocate a proportional cost, such that the largest amount should be paid by the person contributing most to the energy cost, while the person who did not consume any energy should pay nothing. Similarly, in the context of this work, a group of neuronal elements should be ranked fairly according to their contribution to the performance of the network. In fact, Shapley values do so by mathematically satisfying the following axioms [23]:

1. **Symmetry:** If two elements are functionally interchangeable, then their contributions will not differ by changing their labels.
2. **Null player property:** If an element does not contribute to the given function, its Shapley value is zero.
3. **Additivity:** Summing the contributions of all elements results in the outcome of the grand coalition, *i*.*e*., the network’s overall performance.

**Fig 3:**
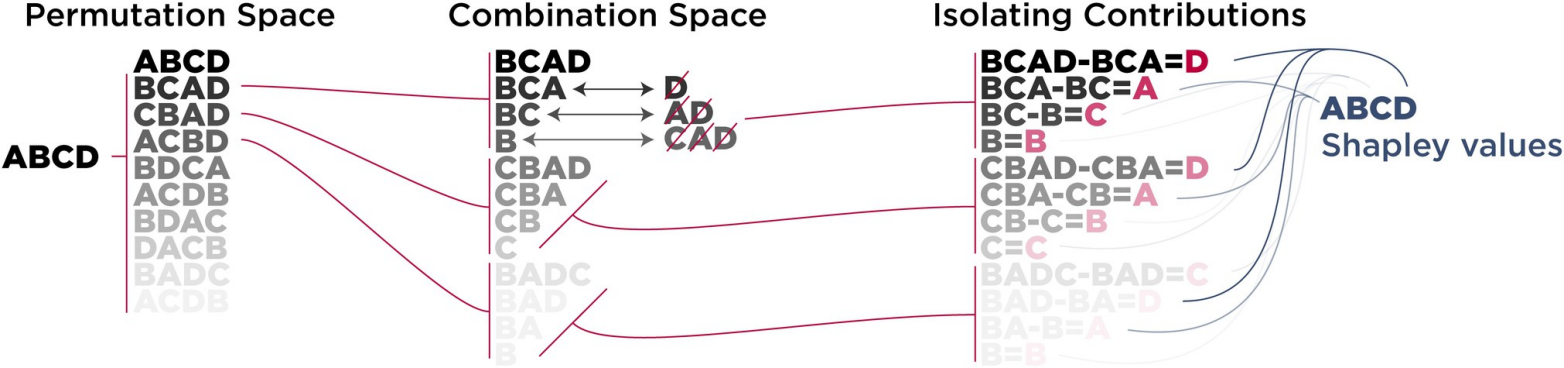
Visual depiction of the MSA algorithm. Since there are 2^N^ possible combinations of coalitions, an analytical solution for the Shapley value of elements in large systems is computationally prohibitive. Therefore, we sampled 1,000 permutations from all N! possible orderings (permutation space) and used them to dictate which coalitions to perturb (combination space). The contribution of elements to each coalition was then quantified by calculating the difference between the score of the coalition with the element (e.g., {B, C, A}) and the score of the same coalition without the targeted element (i.e., {B, C} to isolate A). Shapley value of each element was then calculated by averaging individual contributions of each element to the formed coalitions.

As with the SPA framework, MSA aims to find elements that, when lesioned, most strongly impair the behavior. In this case, these elements have the highest Shapley value derived from their role in all combinations of multi-site lesions, rather than isolated single-site lesions. To elaborate on the pipeline for computing Shapley values, first the individual elements are shuffled iteratively to form a permutation space. Then, for each permutation, a set of elements excluding the target element is lesioned and the performance of the lesioned system is quantified. The target element is then lesioned alongside the lesioned set and the performance is quantified again. The difference between performances in these two conditions, both negative and positive, is what lesioning the target element contributed to that specific group of lesioned elements (Fig 3). Lastly, averaging over these contributions results in the Shapley value of the target element, indicating its average marginal causal contribution to the system’s performance.

Exploring all possible combinations of subsets can be computationally prohibitive in large sets. Therefore, we used an unbiased estimator of the Shapley value that samples coalitions from the space of 2^N^ possible combinations, where N is the number of all elements. Here we used 1000 permutations per element, since we found that the resultant Shapley values were consistent with those estimated from a larger number of permutations. However, this is not necessarily a representative case and exploring larger permutation spaces might yield more stable rankings for some settings (see [22] for more details).

As mentioned in the third axiom above, the Shapley value is additive and, thus, has an intuitive interpretation in which sum of all values results in the performance of the intact network, which here was 337. This means that an element with a Shapley value of 80 accounts for a fraction of 23% of the network’s performance. A negative Shapley value follows the same line of interpretation, that is, an element with a Shapley value of -80 on average prevents the network from an additional 23% increase in performance.

As depicted in Fig 2B, MSA indicates many noncontributing nodes and connections, just as the single-site lesion analysis did. Importantly, according to the MSA, neuron 0 is the most influential, followed by many less critical nodes. Interestingly, neuron -4 is assigned a negative Shapley value, indicating its proportionally large and disruptive contribution to the intact system. This result contradicts the result obtained from SPA that pointed to nodes -5 and 4 to have such an influence (Fig 2C).

As with SPA, we also perturbed and analyzed connections but this time using MSA, to test if we could further trace the causal influence of critical neurons down to their connections. Again, we speculated that either no particular single connection would have a disproportionately large Shapley value, or there were critical connections corresponding to the influential nodes.

MSA traced the importance of node -4 to a single connection from -4 to 0, and similar observations applied to the elements with the highest Shapley value. For example, the causal contribution of neuron 0 could be attributed to its connection (0 → 0), since, apart from (0 → 0) and (-4 → 0), other connections of this neuron had negligible contributions (Fig 2B). As a sanity check, we performed the same procedure on the blinded network. Here we expected that no element would contribute to the network’s overall performance, since, on average, the network had the same baseline score. As shown in S2 Fig this was indeed the case.

The most crucial difference between SPA and MSA was how the respective approaches ranked connections (0 → 0) and (-4 → 0). Remember that even the SPA indicated (0 → 0) as the most critical connection, while perturbing all 11 connections of node 0, including (0 → 0), resulted in no adverse effect. MSA attributed a negative Shapley value to the connection (-4 → 0), meaning that, on average, lesioning (-4 → 0) *alongside other elements* resulted in an improvement of performance. Interestingly, SPA assigned minor importance to (-4 → 0). This means that removing (-4 → 0) *alone* resulted in no particular change in the performance.

Altogether, MSA and SPA found key elements to be a small and localized set. MSA differentiated these results and assigned the negative contribution to neuron -4, which SPA missed. While SPA excluded neuron 0, MSA ranked it as the most critical neuron and further dissected this importance to the self-loop. It then assigned a negative value to an incoming connection from -4 to 0 that could explain why removing node 0 with all its connections did not impact the behavior, while removing its feedback loop alone did. We further dissect the relationship of these connections in the section Understanding the Paradoxical Lesion Effect.

### Impact of Lesioning on functional connectivity

**Fig 4:**
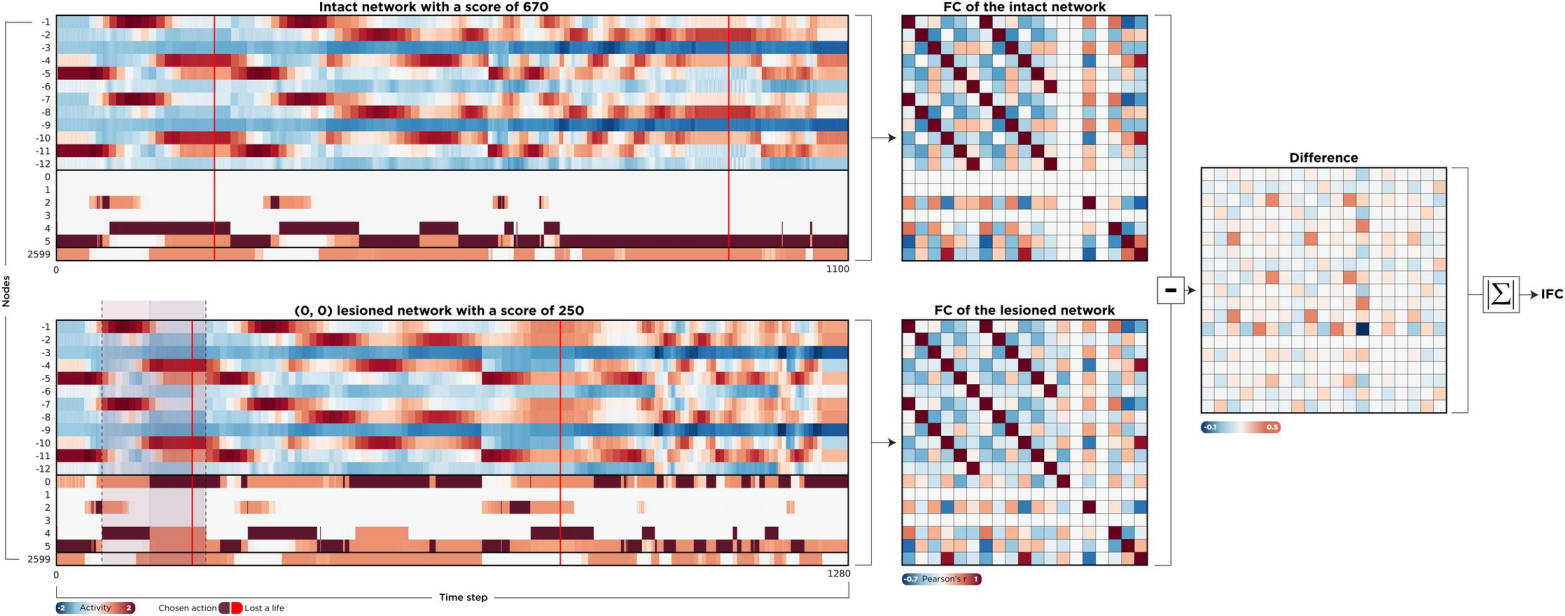
Calculating the impact of lesions on functional connectivity. We recorded the activity of all neurons to compute the functional connectivity of the network. We exhaustively perturbed all units one by one and compared the element-wise differences between intact and lesioned FC matrices. The absolute sum of this difference matrix (IFC) quantifies how much a lesion caused the network dynamics to deviate from the uninterrupted state. On the left-hand side, the activity of two scenarios is depicted. In the upper timeline, the network is intact, and the score is 670, while in the lower timeline, the feedback loop (0 → 0) is lesioned, leading to a drastic decrease in performance. Red vertical lines show when the agent was shot and lost a life. Brown cells indicate the chosen action, and the dashed window is the same time window that was zoomed in in the section *Understanding the Paradoxical Lesion Effect*.

In addition to their direct impact on the behavioral functions of a system, lesions may also disrupt the distributed activity patterns and functional connectivity (FC). In turn, different features of the impact on FC are associated with behavioral performance. Thus, FC forms a bridge, or ‘intermediate phenotype’ from structure to function and behavior [30–33]. It was shown that lesions of critical brain regions in terms of FC, such as hubs, have a greater impact on the dynamics of the whole brain [32]. To explore this aspect in our *in-silico* model, we first calculated the FC of the intact network using Pearson’s correlation of the nodes’ time-series. We then employed a SPA framework for all units, that is, nodes and connections. To quantify the impact of lesioning individual elements on global FC, we calculated the element-wise differences between intact and lesioned FC matrices. The absolute sum of the resulted difference matrix was considered as the impact of lesioning on Functional Connectivity (IFC; Fig 4). A larger IFC results from a greater difference between FC of the intact network and FC of the lesioned network and intuitively indicates the importance of elements, this time by their contribution to overall functional connectivity, instead of performance.

**Fig 5:**
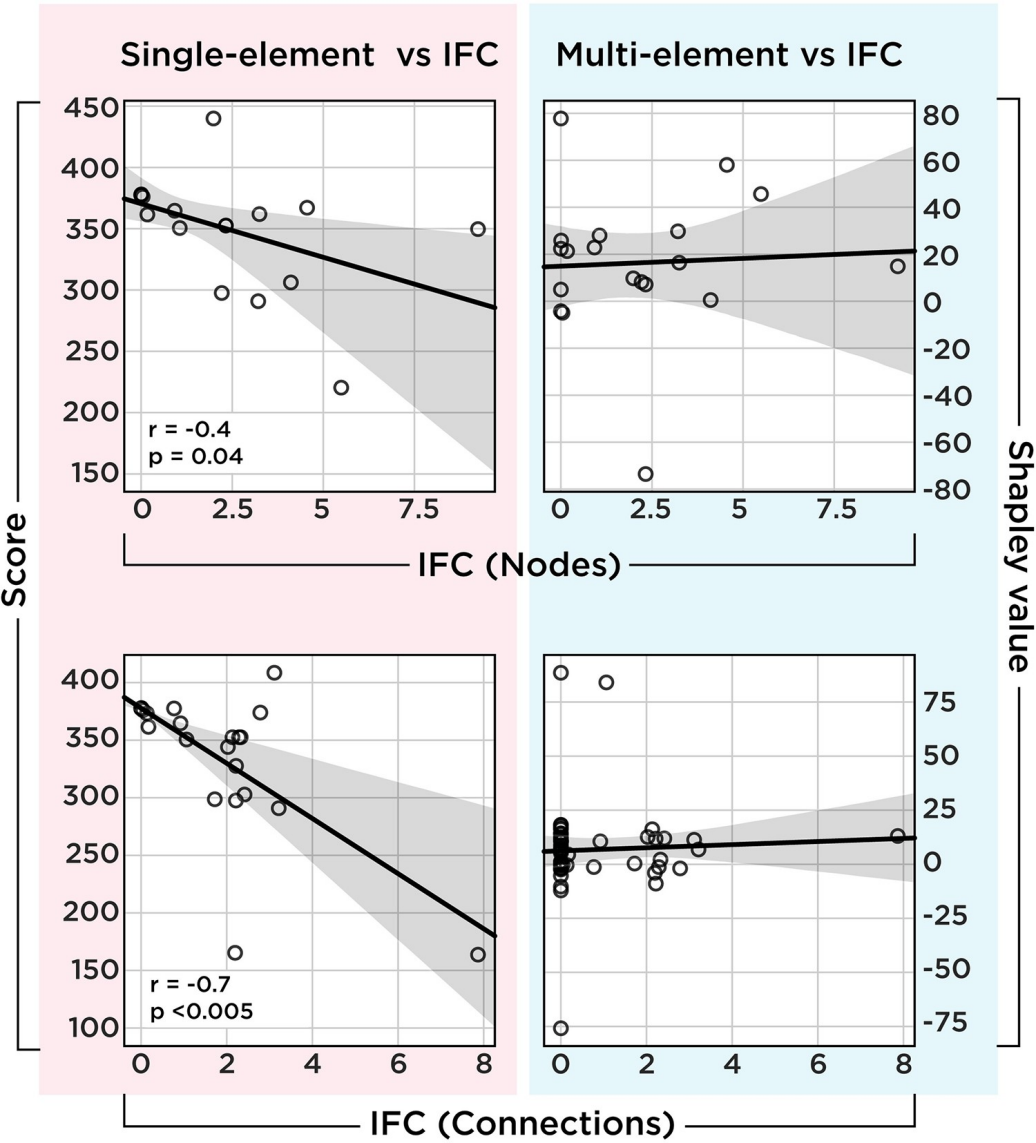
Correlation between IFC and single-site lesioning scheme. The upper left scatterplot shows the relationship between the impact of the SPA of nodes on functional connectivity and the agent’s performance. The lower left scatterplots show the same relationship but for each connection. Both show a negative correlation, which means that the larger the impact on functional connectivity, the lower the performance. However, this relationship is absent from the right-hand side that compares the Shapley value of each element with their IFC. As with the left-hand side, the x-axis shows the IFC of nodes (upper plot) and connections (lower plots), while here, the y-axis represents Shapley value instead of raw performance.

Interestingly, IFC is negatively correlated with both nodal and connection perturbation scenarios, corroborating previous findings (Fig 5). However, IFC is not associated with Shapley values of these elements. This means that although SPA has internal coherency by identifying units that, perturbed one by one, have the largest effect on both functional connectivity and the agent’s performance, these units are not the same as those captured by an MSA framework. In other words, by employing SPA, changes in the dynamics of the network supports its ranking of critical nodes. However, as shown in Fig 2, the actual players remained obscure. In the next two sections, we show why the rankings differ and propose a possible underlying mechanism that accounts for this discrepancy.

### Quantifying complex interactions between causal building blocks

In previous sections, we presented two causal rankings of elements from the same ground-truth neural network model, one using a SPA framework and the other using MSA (Fig 2C). We found that the changes in the inner dynamics of the system perturbed using SPA support this approach’s ranking, which mistakenly adds more confidence to the accuracy of the approach in finding critical units. Here we show why these rankings differ by measuring the complex interactions of units. Although MSA is a multivariate approach that accounts for a large variety of combinations of units, it eventually describes the system in terms of how much, averaged over all combinations with other units, *single units* contribute to the output. In other words, it isolates the average *individual* contributions, and not directly reveal their interactions by which the causal influence of one element is intertwined with the state of others. Using an extension of MSA, here called Pairwise Causal Interaction Analysis (PCIA) [21], we formalized and then quantified these interactions.

**Fig 6:**
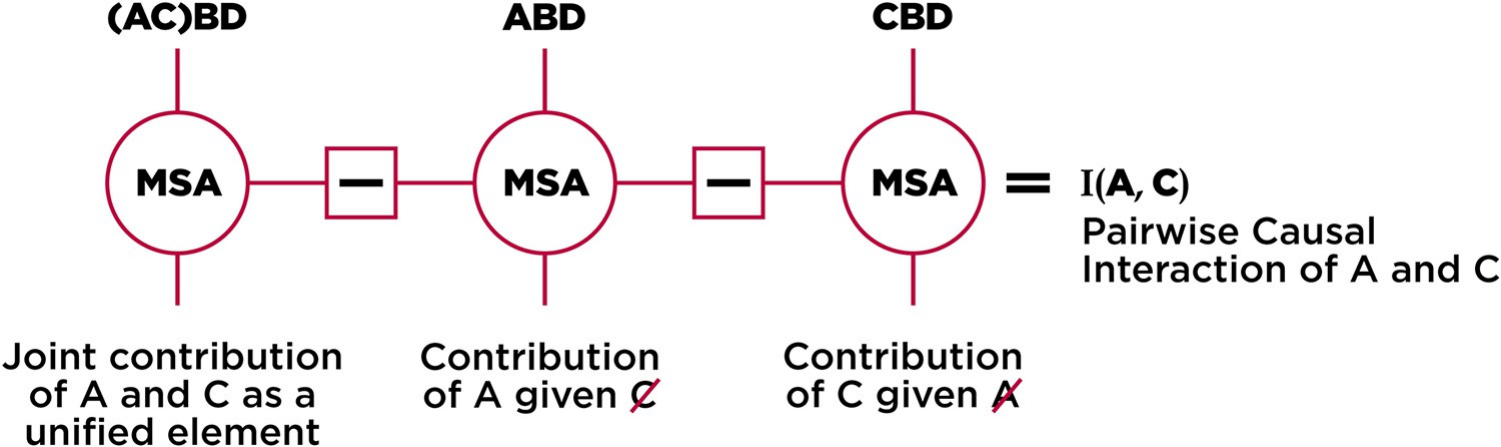
Visual depiction of the PCIA algorithm. At its core, PCIA comprises multiple MSAs. We first start with calculating the joint contribution of two elements, followed by the contribution of each, given the other is perturbed. The interaction term is then calculated by subtracting these values from each other, indicating how much the joint contribution of a pair of elements is bigger or smaller than the sum of their individual contributions.

To elaborate, quantifying the complex pairwise interaction of two elements *i* and *j* in contributing to behavior requires to calculate the Shapley value of them jointly as a single compound element *γ*_(*ij*)_, as well as the Shapley value of each of them separately while the other one is perturbed, $\gamma_{\left(\bar{i},j\right)}$ and $\gamma_{\left(i,\bar{j}\right)}$, respectively. As Fig 6 shows, subtracting the conditioned contributions from the joint contribution provides an interaction term that, if positive, indicates *“net-synergy”* between the pair and, if negative, shows *“net-redundancy”* or functional overlap. Note that the definitions of synergy and redundancy here are not the same as those defined by information theoretical approaches, for example Partial Information Decomposition (see [34]). Conceptually, PCIA quantifies how much the causal contribution of a joined pair of units is bigger or smaller than the sum of their individual contributions [21], with contribution here referring to a quantity *with respect to the networks performance*. In other words, PCIA quantifies the difference between the joint contribution of *i* and *j* and their individual contributions to the performance.

Since PCIA involves the calculation of multiple MSAs, it is computationally even more expensive than the MSA of individual elements. Therefore, we focused on the connections, and to calculate all pairs of them, we sampled 100 permutations per element instead of 1000, as in the case of MSA.

**Fig 7:**
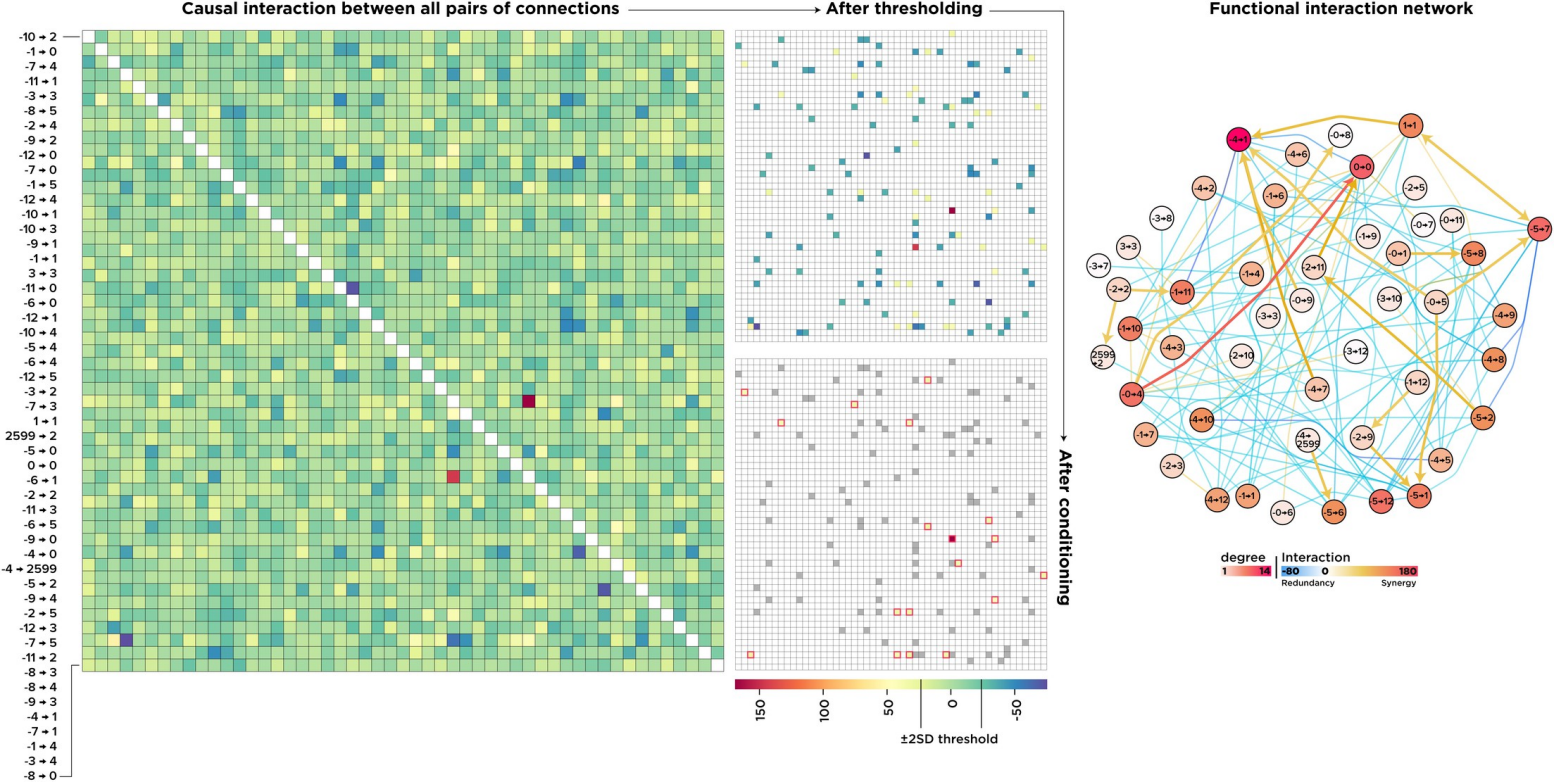
Pairwise interactions among all connections. An interaction matrix resulting from the PCIA procedure in which warmer colors show greater synergy and cooler colors indicate functional overlap (left). We excluded the values within a range of ±2 SD above and beyond the mean and applied the “Sprague effect” condition to the thresholded matrix (middle). On the right-hand side, we plotted the interaction network in which the nodes represent connections in the actual network, and the edges are interactions among them. Arrows show paradoxical-lesion effects (i → j).

The results are shown in Fig 7, and as quickly stands out, there appeared a strong net-synergy between two elements (0 → 0) and (-4 → 0), followed by a handful of strongly redundant and many minuscule interactions in both directions. We next formalized the Sprague effect as a scenario in which element *i* has a negative Shapley value when element *j* is perturbed $\gamma_{\left(i,\bar{j}\right)}<0$, thus hindering the performance and has a positive contribution when *j* is intact $I_{i,j}+\gamma_{\left(i,\bar{j}\right)}>0$. Put simply, on average, element *i* disrupts the performance if element *j* is intact and improves if *j* is lesioned [22,25].

To reduce the number of false-positive findings, we looked for this condition among a smaller set of pairs with an interaction term above and below two standard deviations of the mean. The results are shown in Fig 7, with connections indicating the interactions and arrows depicting a Sprague effect between two elements (the stem of the arrow indicates the element *i* that has a negative contribution when the pointed element *j* is lesioned.) As depicted, we found many paradoxical lesion effects predominantly among synergistic interactions, with the interaction between (0 → 0) and (-4 → 0) being the most prominent one. Note that the network in Fig 7 is a higher-order “interaction network” in which the nodes represent connections in the “structural network”.

To summarize this part, we first quantified how much the joint causal importance of two elements is larger or smaller than the sum of the importance of the individual elements. We then found a handful of elements in which lesioning one, while the other one was perturbed, restored the performance. Among them, the connections (0 → 0) and (-4 → 0) had the highest net-synergy, meaning that, jointly, they functionally contributed much more than their summed individual contributions. This unique interaction is also a paradoxical-lesion effect in which lesioning (0 → 0) alone disrupted the performance while lesioning it alongside (-4 → 0) restored it.

In the next section, by focusing on the two connections (0 → 0) and (-4 → 0), we show that paradoxical lesion effects are not an uncommon phenomenon and that their frequent occurrence might be a direct result of perturbing a simple and ubiquitous motif of connectivity.

### Understanding the paradoxical lesion effect

Paradoxical lesion effects, such as the Sprague effect, were first described in cats and later in humans, with the underlying mechanisms still partly elusive [18,19]. One theory suggests that the phenomenon is caused by a reduction of inhibition from a functionally competing region, and the deficit reverses when both regions are lesioned [35]. To see if this is the case in our network, we focused on the two most prominent units (0 → 0) and (-4 → 0). Note that the SPA also ranked (0 → 0) among the most critical connections. However, (-4 → 0) was only captured by MSA and was the only unit with a large negative Shapley value.

**Fig 8:**
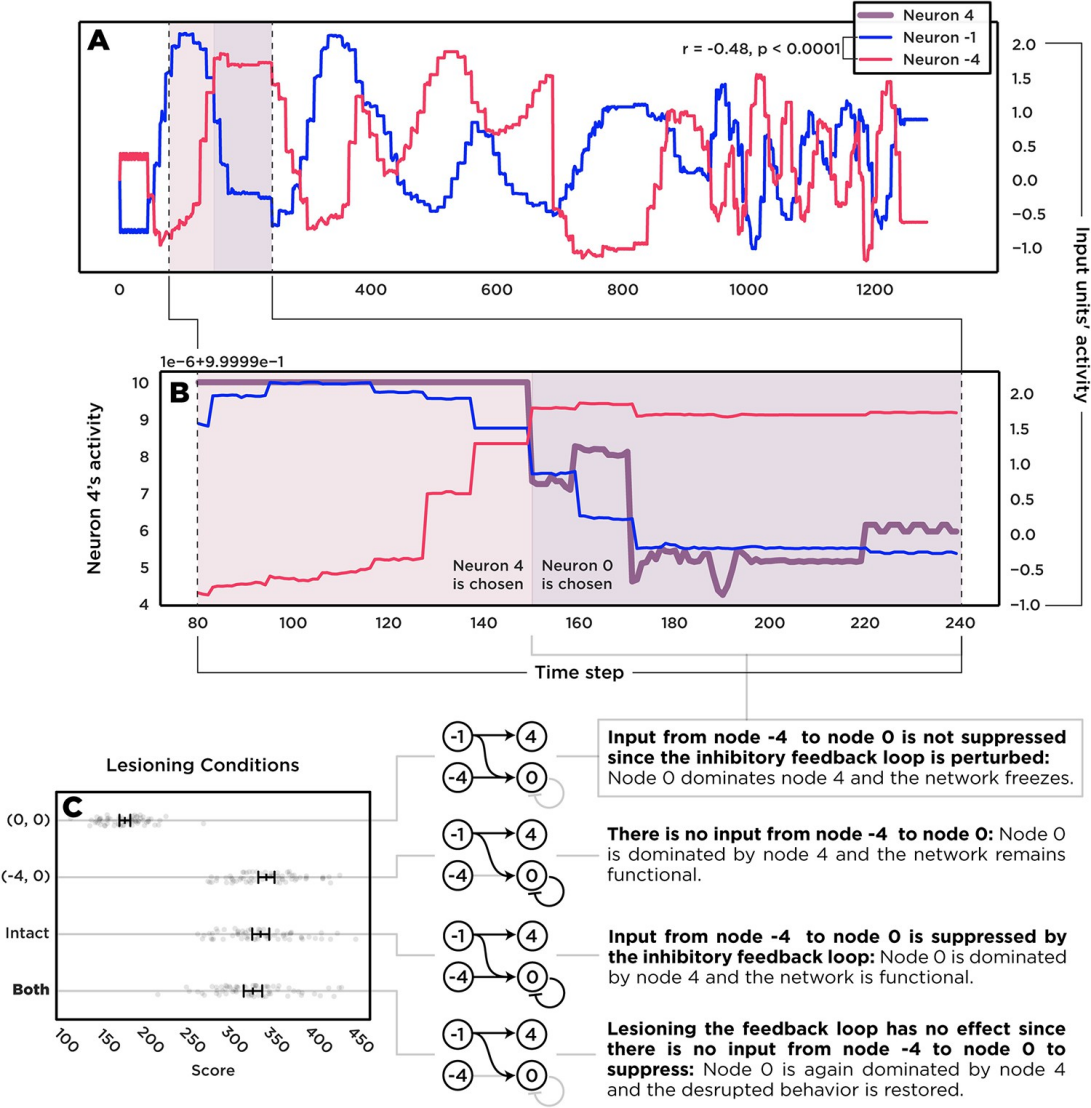
Focusing on the critical elements discovered by MSA. The panel A shows the negative correlation between the activity of two input units -1 and -4. This anticorrelation leads to competition between downstream units 4 and 0. In an intact network, unit 4 is dominant due to the inhibitory feedback loop of unit 0. The panel B shows how activity of unit 4 decays since it is tightly coupled with the input from -1 while neuron 0 without its feedback loop is driven by the input from -4. The panel C shows the implications of this rivalry on the performance and how it produces the paradoxical lesion effect. Lesioning the feedback loop disrupts the performance while lesioning it alongside the input from -4 restores the deficit since neuron 0 stays dominated.

Fig 8A shows the activity of two nodes, -1 and -4, over a trial in which (0 → 0) is lesioned (also see Fig 4). A Pearson’s correlation analysis showed that they are negatively correlated. Node -1 was one of the key inputs to node 4, which itself was one of the most frequently chosen actions by the intact network. Node -4, however, had a strong influence on node 0 (Fig 1) that was inhibited by the negative feedback loop (0 → 0). Since node 0 was the action “no action”, keeping it dominated allowed the intact network to choose other actions and function properly. Fig 4 shows how lesioning the feedback loop disrupted the suppression and led to the hyper-activation of node 0. Fig 8B shows how the decaying activity of node -1 caused node 4’s activity to decay as well and eventually ‘lose’ to node 0 in the lesioned network. Naturally, the behavioral consequence of excessively choosing “no action” was a substantially lower score. These findings explain the difference in rankings from MSA and SPA in which lesioning (-4 → 0) alone had no impact on behavior while lesioning it along other elements improved the performance:

If node 0 received either no input from -4 or was able to suppress the input, then the network remained functional. Removing the feedback loop (0 → 0) alone, however, resulted in the over-activation while removing (-4 → 0) alone was harmless. Perturbing both then resulted in a paradoxical lesion effect because by lesioning the input from -4 to 0 with or without the feedback loop, node 0 never reached the critical threshold to dominate other actions, and thus, the performance was restored (Fig 8).

Altogether, by looking deeper into the inner interactions of the units that MSA distinguished, we see that a simple motif of connectivity among only four units was enough to produce a paradoxical lesion effect. The key nodes were -4 and 0; the key connections were (0 → 0) and (-4 → 0). The input from -4 to unit 0 had a large negative Shapley value because in coalitions without (0 → 0), it over-activated neuron 0 and caused the network to freeze. The feedback loop (0 → 0) had a high positive Shapley value, because it prevented this over-activation and removing it caused the network to freeze. Interestingly, the input from -1 to 4 had the next highest Shapley value because, without it, node 4 was dominated by other units, especially an over-activated “no action” node.

To conclude this section, we showed that a paradoxical lesion effect could emerge from a simple inhibitory motif. In our case, the inhibition was a negative feedback loop, and the competition was between two output neurons, 4 and 0. We show that, even in a simple agent, revealing with confidence which elements are causally relevant for behavior and how is extremely difficult. In the next section, we discuss our results, limitations, and potential future improvements.

## Discussion

In this study, we defined causation as contributions of individual elements to a collectively produced function, and specifically aimed to understand an ANN in terms of the causal contributions of its components to its game-playing performance. We compared two principal approaches for determining the causal contributions, lesioning its neurons and connections *one element at a time* (Single-element Perturbation Analysis, SPA), versus lesioning and analyzing multi-site lesion effects using the multivariate Multi-perturbation Shapley value Analysis (MSA).

We found the causal ranking produced by MSA to be substantially different from the ranking provided by SPA. Interestingly, MSA ranked nodes and connections such that the most critical connections belonged to the most critical nodes, a pattern that was missing from the SPA ranking. Using an extension of MSA, we also quantified the complex pairwise interaction of all connections. Specifically, we identified a pair of connections that, when perturbed, produced a paradoxical lesion effect, where lesions of one connection produced a functional deficit, but lesions of both connections restored function. This phenomenon was underlying the biased ranking of elements by SPA, since, by definition, the effect manifested only after a simultaneous lesion of two elements. Finally, we focused on the two connections that were ranked most critical by MSA. We found a rivalrous interaction between these two units to be the mechanism responsible for the paradoxical lesion effect.

### MSA: A step towards quantifying causal contributions

A substantial challenge in depicting a mechanistic blueprint of the brain is to achieve a comprehensive causal understanding of it. We propose that a step in this path is to quantify *the degree* to which neural elements contribute to cognition and behavior. A knowledge of *how much* the element is contributing to a function allows us to rank those elements accordingly. The Shapley value as a measure of causal contribution is powerful and intuitive, however, it is important to emphasize what this value does not reflect (see [36] for a technical perspective). For example, the Shapley value by default does not reveal mechanisms, in the sense that it does not show what computations were done by individual elements. Instead, it shows *how much each element is functionally contributing to the underlying mechanistic processes*. Thus, this is only the first step towards a comprehensive mechanistic description of the brain, illuminating which elements to focus on. In the present study we did so by focusing on the few key elements with high causal contributions and further investigating their connections and functional interactions.

### Versatility of MSA

In this work, we used the metric of ‘performance score’ since it was relatively straightforward to interpret in the given experiment. However, further analyses could decompose the behavior and calculate the causal contribution of the elements to each behavioral component. Therefore, we can expand our knowledge of how elements dynamically form coalitions to solve sub-tasks of the given task [37]. MSA is a robust method to unravel how neuronal units adaptively join communities and produce functional hierarchies in the brain.

Additionally, MSA can incorporate metrics and concepts that are already developed to infer causal structure in networks [38]. Here, we used a different definition for the concepts of net-synergy and net-redundancy than those provided by the partial information decomposition approach [34]. These concepts can be harmonized by tracking the synergistic/redundant contribution of each element to the permuted coalitions instead of its contribution *per se*. Namely, if the contribution of coalition {A, B} is 10, and the contribution of {C} is 5, then the expected contribution of {A, B, C} is 15. However, if adding {C} to {A, B} resulted in values larger than 15 then the contribution can be assumed net-synergistic, and net-redundant if it is smaller than 15.

We also did not pursue the relationship between information and causal contributions here. In fact, one can unify information theoretical concepts by tracking, for example, entropy instead of the performance score. Another possibility is to lesion the information by injecting noise instead of perturbing the node itself [39].

Moreover, we used MSA to compute causal contribution of nodes to behavior, which means that the causal relationships within the system, that is, the causal contribution of nodes to other nodes, remained opaque. One can infer such relationships by iterating MSA on each node while computing causal contributions of others to it. For instance, and to bridge MSA to information theoretical approaches to uncover causal relationships in the network, one can iteratively perform MSA on each node and track the changes in, *e*.*g*., the mutual information of the pre-and-post perturbation signals produced by the target node *i*. This way, MSA ranks the nodes according to their contributions to *i*, such that lesioning the most critical node *k* results in a reduction of ‘self-similarity’ in *i*, implying a large deviation of *i* after perturbing *k* compared with the activity of *i* before perturbing *k*. Generally, the MSA approach is versatile and can be adapted to track many metrics and be incorporated in diverse analytical pipelines. The present work, however, had a more specific agenda and further research is needed to establish pipelines integrating already existing metrics with MSA.

### Current limitations of MSA

Although conceptually and methodologically powerful, MSA has limitations that need to be addressed. The main limitation is MSA’s computational complexity. Reaching an analytical solution for Shapley values of large systems is an NP-complete problem [40]. Therefore, heuristics [41], predictors [24], and estimators [22,42] are used and are under further development.

Another current limitation that requires future investigation is the implementation of a pipeline for higher-order causal interaction analysis. Here we used a pair-wise approach (*i*.*e*., PCIA; [21]), and it would be interesting to quantify the complex interaction of elements in higher dimensions. Two points need to be addressed, first the computational complexity and second the complexity of the results, as PCIA is already accounting for higher-order perturbations, which makes it difficult to interpret the findings of extensions beyond PCIA. Additionally, in this work, thresholding the ‘interaction matrix’ (Fig 7) was done arbitrarily. To decrease the computational cost of the analysis, we reduced the number of samples from 1000 to 100, which means less certainty in the estimated results. To partly account for this problem, we excluded values within the range of two standard deviations above and below the mean, a decision that directly influences the number of discovered paradoxical-lesion effects. Therefore, a central interest is to address this issue using either better thresholding criteria or estimation methods.

### Paradoxical-lesion effects and their implications

Paradoxical lesion effects are intriguing functional phenomena that have attracted much attention and controversy [43,44]. How were paradoxical lesion effects produced in our example network? First, our network was fixed throughout the experiments. This setting leaves no room for plasticity. Second, the network was a simple ANN with no excitatory-to-inhibitory synaptic dynamics. It is possible that such physiological mechanisms underlie paradoxical lesion effects in the living brain [19]. However, we did not include them in our model; therefore, the paradoxical effects observed here do not result from such dynamic mechanisms. We found functional inhibition between competing units sufficient to produce a Sprague effect, as also investigated before ([35,45]; cf. [25] for a fixed artificial network). Here we observed paradoxical lesion effects in a network of 19 neurons, therefore, further research is needed to estimate the possible number of connectivity motifs that can give rise to paradoxical lesion effects in larger networks. Also note that the motif was costly and sub-optimal, because instead of simply removing the over-activating input, the evolutionary process added a negative feedback loop to cancel its disruptive influence, which produced the motif and led to a paradoxical lesion effect. It remains to be investigated if other types of connectivity motifs can also give rise to paradoxical lesion effects.

### Single-site lesion analysis as a framework for testing hypotheses

Given our findings, we suggest that SPA by itself does not provide definitive conclusions on the causal contributions of system elements(cf. [14]) and if used, it needs to be combined with clear hypotheses and supported by other methods. For instance, Inagaki and colleagues [46] showed that during a delayed response task, the anterior lateral motor cortex of mice shows a persistent activity that was involved in maintaining short-term memories. Using perturbations, they revealed that the activity of neurons in this region moves towards discrete dynamical regimes to direct the decision and is generally robust against perturbations. However, perturbations occasionally resulted in switching between these regimes that consequently caused a wrong decision. Hence, they combined SPA and computational modeling to answer *what part of the computation* a target region might be involve in [46]. The point of this study is the fact that SPA is used alongside other methods to test a hypothesis, not to map the short-term memory to the anterior lateral motor cortex as such. To summarize this aspect, and considering paradoxical lesion effects and the fact that SPA is partially insensitive to identifying the causal contribution of the involved elements, we suggest that SPA approaches should be used less as a brain mapping tool and more as a hypothesis testing tool. Even used as such, the results should be interpreted cautiously and contrasted with other approaches.

### Potentials of *In-Silico* experiments and ground-truth models

Finally, in this work, we used a version of Evolutionary Autonomous Agent models advocated by [47] as a helpful simulation environment for neuroscientists. Using NEAT, we allowed the network’s topology to evolve with respect to the environmental constraints instead of modeling the architecture ourselves and optimizing the weights or readout units. This way, we liberated ourselves from further assumptions about the network’s connectivity and structure. We see a potential role for such algorithms in neuroscience since one can evolve arbitrary architectures to solve an ecologically valid task, such as foraging in a patchy environment [48], and compare their topological features with brains evolved in similar environments.

**Fig 9:**
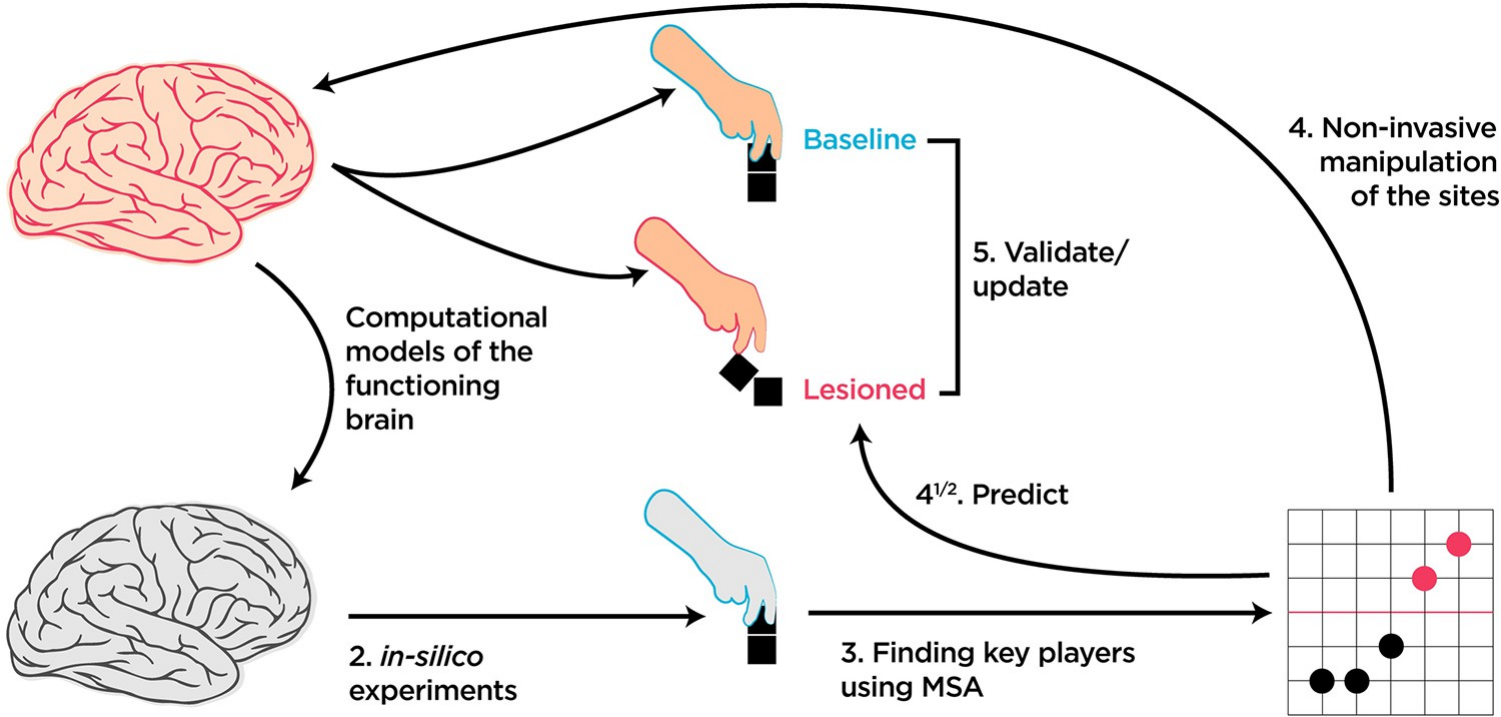
How MSA can be incorporated into the causal brain-mapping toolbox. Since exhaustive multiple brain lesioning in experimental biological research is currently beyond the reach, we suggest connectivity-aware or neural network models of functioning brains to fill the gap. *In-silico* experiments can then be performed to predict key elements and their contributions to the behavior. These predictions can then be tested experimentally *in-vivo* by the method of choice.

More cognitively and clinically oriented, *in-silico* multi-element lesioning experiments can be used as predictive tools to guide non-invasive brain stimulation experiments. For example, human brain connectivity can be used as the backbones of ANNs trained to solve cognitive tasks [49–52]. These connectivity-aware ANNs can then be investigated thoroughly using MSA to predict critical regions and connections and the corresponding behavioral deficits. The predictions can be used as testable hypotheses about which regions to perturb *in-vivo*. In other words, brain-inspired ANNs, neural network models of cognitive processes [53], and large-scale models of functioning brains [54] can add a unique value to the repertoire of ground-truth models to test brain-mapping tools and their limitations (Fig 9).

## Materials and methods

In this section, we explain the methods and materials used in this research. The codes and generated datasets are publicly available in the following repository:

https://github.com/kuffmode/ANNLesionAnalysis

Briefly, we first trained a deep autoencoder to compress the screen pixels to a handful of features per frame. We then evolved a controller network to, based on these features, choose a proper action. After having both networks, we started the lesioning experiments.

### Evolutionary optimization

We used the NEAT-Python toolbox [55] to evolve a network from an initial stage of randomly connected 12 input and six output nodes. During the evolutionary process, the algorithm was optimizing many parameters, including the choice of activation functions, aggregation functions, adding or removing hidden neurons, adjusting connection weights and node biases, and adding or removing connections (see Table 1 for a summary and the file AEconfig-SI.txt for the complete list of hyperparameters). There were no restrictions on the connectivity pattern so that a recurrent architecture could evolve from the initial feed-forward stage. We chose the probability for removing connections to be slightly higher than for adding (0.6 versus 0.5) to encourage sparsity. We then ran the evolutionary processes 32 times to have 32 candidates. The process ended either after 128 trials or when one member reached the fitness criterion of 1200 points. In each trial, the generation comprised 128 members that were instantiated from the same initial stage and would play the ATARI game independently. After each step, the algorithm mutated the genome according to the given probabilities and performed the cross-over among the top %30 networks to produce the next 128 members. At the end of the training phase, 32 candidate networks reached either the generation limit or the fitness criterion. We then chose the network with the highest score of 1300 points to move forward with the lesion experiments.

**Table 1:** A summary of relevant NEAT hyperparameters. NEAT produces a large variety of networks, all from a set of constraints and probabilities. Since our goal was to produce a good-enough network, we did not tune these parameters for maximum performance and either used the default values or adjusted them according to the experimental objectives, e.g., sparse connectivity.

NEAT Hyperparameters	Value
Fitness Threshold	1200
Population Size	128
Activation Function’s Mutation Rate	0.05
Aggregation Function’s Mutation Rate	0.05
Probability of Linking Nodes	0.5
Probability of Removing Links	0.6
Probability of Adding Nodes	0.6
Probability of Removing Nodes	0.4
Number of Input Neurons	12
Initial Number of Hidden Neurons	0
Number of Output Neurons	6
Survival Threshold	0.3

### Preprocessing steps and the autoencoder

We used OpenAI Gym [26] ATARI environment as our game environment. The game screen generates an array with the size of (210, 160, 3); since the screen is 210 × 160 pixels, each contains three color values, red, green, and blue. Throughout the whole work, the pixels passed through a preprocessing pipeline first that would:

1. crop-out the unrelated parts of the screen such as scores and the ground,
2. convert colors to the monochrome gray-scale, therefore reducing the 3D space of red, green, and blue values to one intensity value representing brightness of each pixel,
3. binarize the pixel values to either an *“on pixel”* or *“off pixel”*,
4. and finally, flatten the outcome into a vector with a size of 2679 pixels.

**Fig 10:**
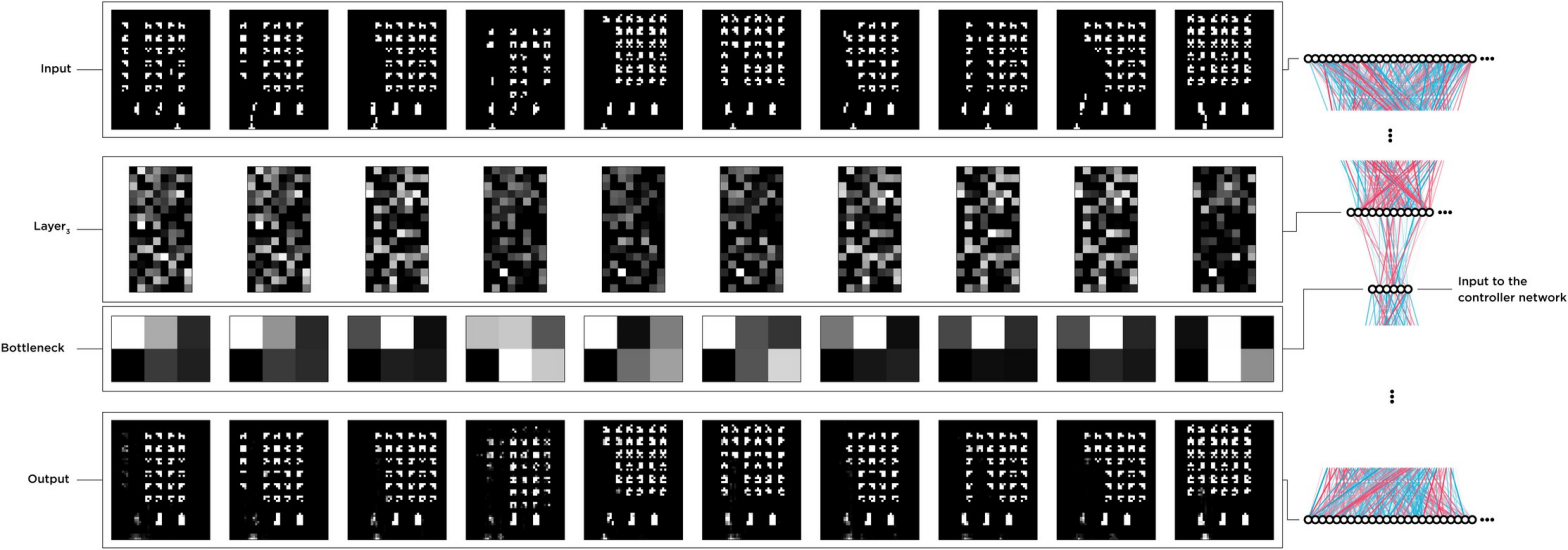
Visualization of the Autoencoder’s inputs, latent features, and decoded outputs. The Autoencoder was trained separately from the controller and received recorded frames from a random action selector agent. We then used the encoder half to reduce the pixel space to six features per frame and fed the controller with two feature vectors.

This vector represented the game with a series of zeros and ones that were then fed to the Autoencoder (Fig 10). The Autoencoder was a Keras model [56] trained independently from the controller network. We first recorded 43,200 frames from the game played by a random agent, shuffled the frame orders, and used 28,800 frames (≈%65 of the dataset) to train and the rest for testing the Autoencoder. The architecture was designed with four encoding layers and four decoding, and a bottleneck of six features.

We used the ADAM optimizer, a binary cross-entropy loss function, 64 epochs, and a batch size of 512. Since input frames are binarized, we used Rectified Linear Unit (ReLU) activation functions for all layers except the last decoding layer, for which we used a Sigmoid function instead. After the training session and accuracy of ≈%98.8, we kept the encoder network and fed the latent space to the controller network throughout all experiments and the evolutionary process of the controller (Fig 10). Together the Autoencoder and the controller network formed our agent. However, we did not perturb the Autoencoder and focused solely on the controller during the experiments.

### Lesion analysis

We first pruned our network by removing the already “disabled” connections. Briefly, connections in the network are either enabled, meaning they multiply the incoming value with the weights and pass it to the receiver node, or disabled and pass zero. During the evolutionary process, these disabled connections serve as “pseudogenes” that can be reactivated in later generations due to mutations. Initially, the controller had 7 of such disabled connections that, after pruning, we had 51 enabled connections to target. We used the same attribute to lesion the connections by virtually disabling them from passing values from source neurons to receivers. In other words, a lesion in our experiments was represented by a severed connection which, technically, would disrupt the flow of information from the source node to the receiver node. To lesion nodes, we then disabled the incoming/outgoing connections. For example, to lesion a neuron that sends information to three other neurons, we set those three connections to zero, which virtually silenced the node.

Each lesion experiment started with silencing the targeted neuron or connection as described. All experiments consisted of 512 trials in which the network played the game 16 times per trial. The score of each trial was calculated by averaging these 16 scores, leading to a distribution of 512 scores per lesion experiment.

### Multi-perturbation shapley value analysis

MSA is a rigorous method based on a game-theoretical metric called the Shapley value, here *γ*, that quantifies the importance of an element for the grand coalition of all elements of a system. To elaborate, assume the marginal importance of an element *i* to a set of elements *S*, with *i*∉*S* is:

$$\Delta_{i}(S)=v(S\cup \{i\})-v(S)$$

with *v* being the worth or importance of the element *i*, and *S* a coalition of elements. Then *γ*_*i*_ with *i*∈*N* is defined as:

$$\gamma_{i}(N,v)=\frac{1}{n!}\sum_{R\in R}\Delta_{i}(S_{i}(R))$$

where R is the set of all n! orderings of *N* and *S*(*R*) is the set of elements preceding *i* in the ordering *R*. We estimated *γ* of all neurons and connections by sampling 1000 sequences from the permutation space of 19! neurons and 51! connections. These 1000 permutations then dictate which combinations of elements should be lesioned (Fig 3). After selecting the target elements, we used the same perturbation approach as for the single-site lesions and disabled the corresponding connections. The agent played the game 16 times, and the average score was taken as the score of that random permutation, providing a *γ* distribution of 1000 data points for each element. Altogether, we had around 70,000 unique combinations of lesions to estimate *γ* from.

### Statistical inference

Besides testing the performance of the intact network against the random agent, blind, and weight-shuffled networks in which we used the non-parametric Mann-Whitney U test, we used bootstrap hypothesis testing to find significant statistics throughout the study. We first generated a synthetic null distribution for each statistical test by shifting the original distribution towards the H_0_’s mean value, either zero or an arbitrary number. For instance, to compare a distribution against a null distribution centered around zero, such as Shapley values, we subtracted the average from each data point, centered synthetic distributions around zero. In cases in which we tested distributions against a second distribution that is not centered around zero, such as the performance of the single-lesioned network versus the performance of the intact network, we shifted the synthetic distributions toward the H_0_’s mean, in this example, around 337 by adding the mean to each data point.

**Fig 11:**
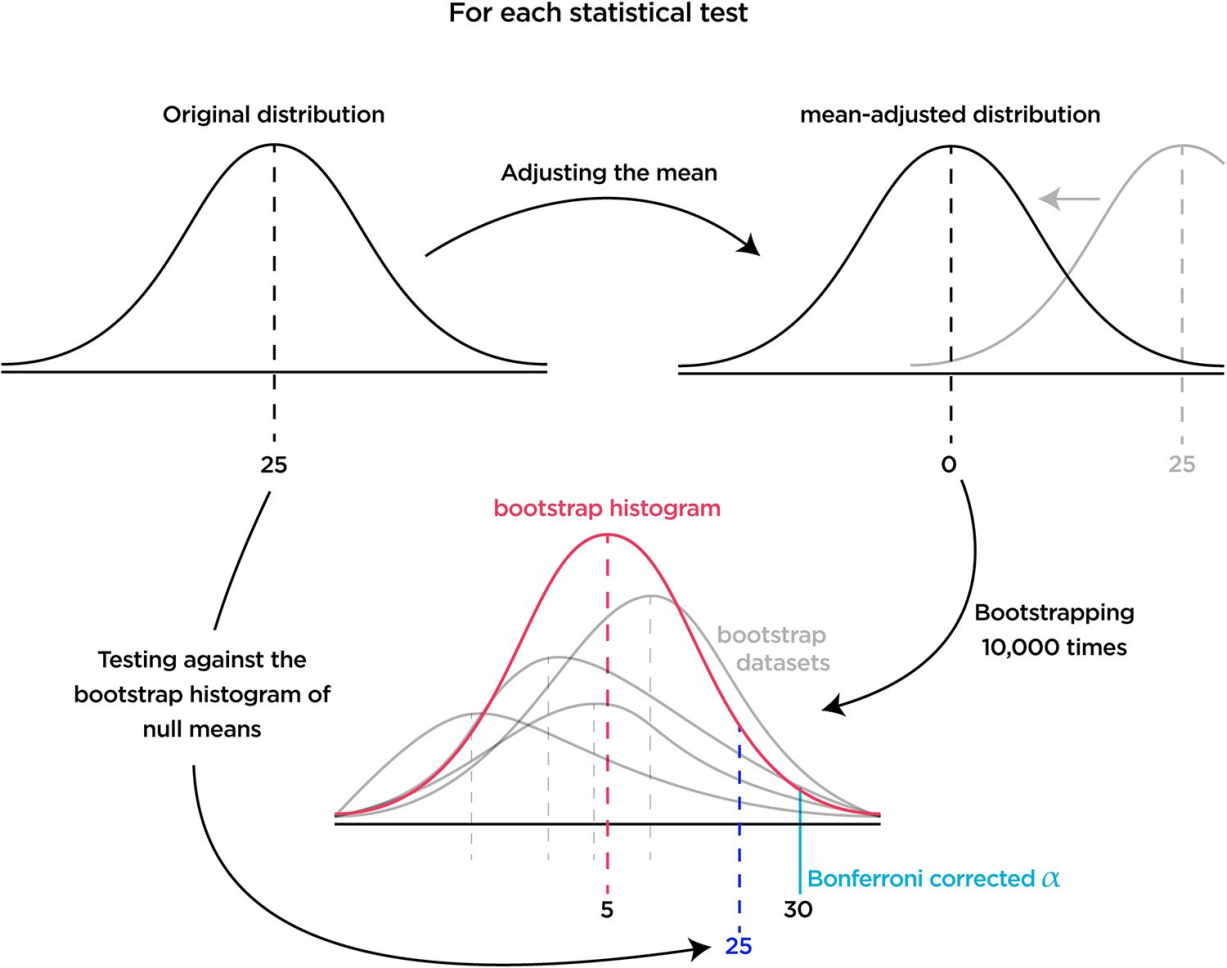
Visual diagram of the hypothesis testing process. For each test, we first created a null distribution by adjusting the mean. Then we resampled the synthetic distribution and kept track of the averages in the bootstrap histogram. Lastly, we checked if the original mean falls below or above the Bonferroni corrected p-value.

We then performed the bootstrapping and resampled the mean-adjusted distributions N times with replacement, with N being the number of original samples, e.g., 512 for single-site lesions. This generated a bootstrap dataset centered around the H_0_’s mean (Fig 11). We then calculated the bootstrap dataset’s mean and repeated the process 10,000 times to generate the bootstrap histogram of the means. In other words, the bootstrap histogram is a distribution of means if they were from a null hypothesis. We then checked if the mean values of our distributions fall above or below the p-value that is corrected for multiple comparisons using the Bonferroni correction method (0.05/Number of tests).

## Supporting information

S1 Fig Distribution of performances. Optimized network is the evolved network, which reached a good-enough performance. Noise-as-input is the same network that receives random values drawn from a uniform distribution [0, 1] as input instead of receiving game-states. Weight swapped network receives the game-states while the connection weights are shuffled. Finally, Random action selector is an algorithm that selects a random action, at each timepoint, regardless of the game-states.
(TIF)

S2 Fig Shapley Values of the blinded network. As a sanity check, we performed the MSA on the optimized network connections while feeding it noise instead of game-states. The procedure is explained in the section: *Multi-perturbation Shapley value Analysis*. We found no connection with considerable causal importance since the network cannot perform properly.
(TIF)
