# Supplementary figures and images for: Systematic perturbation of an artificial neural network: A step towards quantifying causal contributions in the brain
Source: PLoS Comput Biol. 2022 Jun 17;18(6):e1010250. doi: 10.1371/journal.pcbi.1010250 (PMC9246164; doi:10.1371/journal.pcbi.1010250)

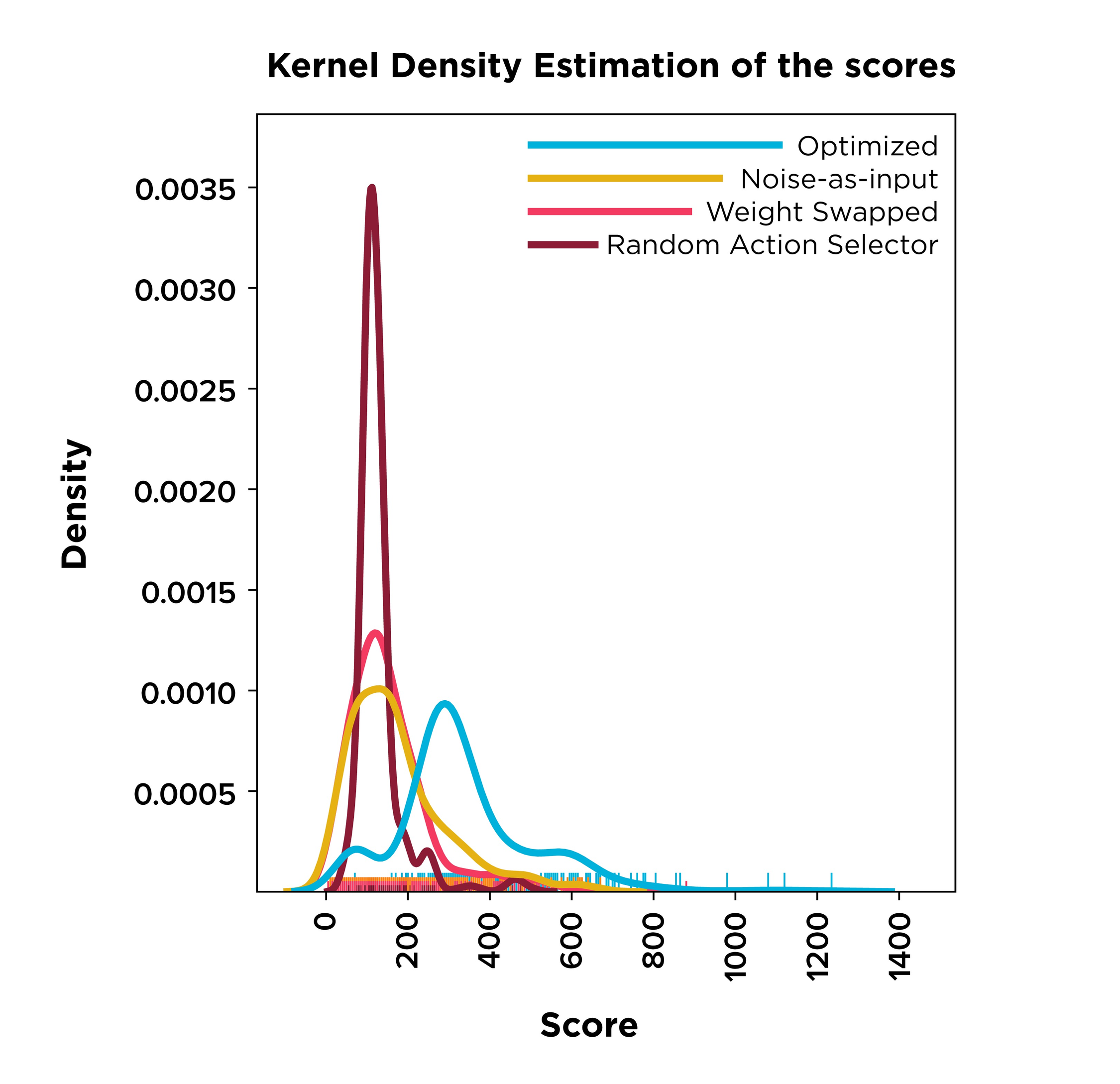

Supplement: S1 Fig — Optimized network is the evolved network, which reached a good-enough performance. Noise-as-input is the same network that receives random values drawn from a uniform distribution [0, 1] as input instead of receiving game-states. Weight swapped network receives the game-states while the connection weights are shuffled. Finally, Random action selector is an algorithm that selects a random action, at each timepoint, regardless of the game-states. (TIF) [file pcbi.1010250.s001.tif]

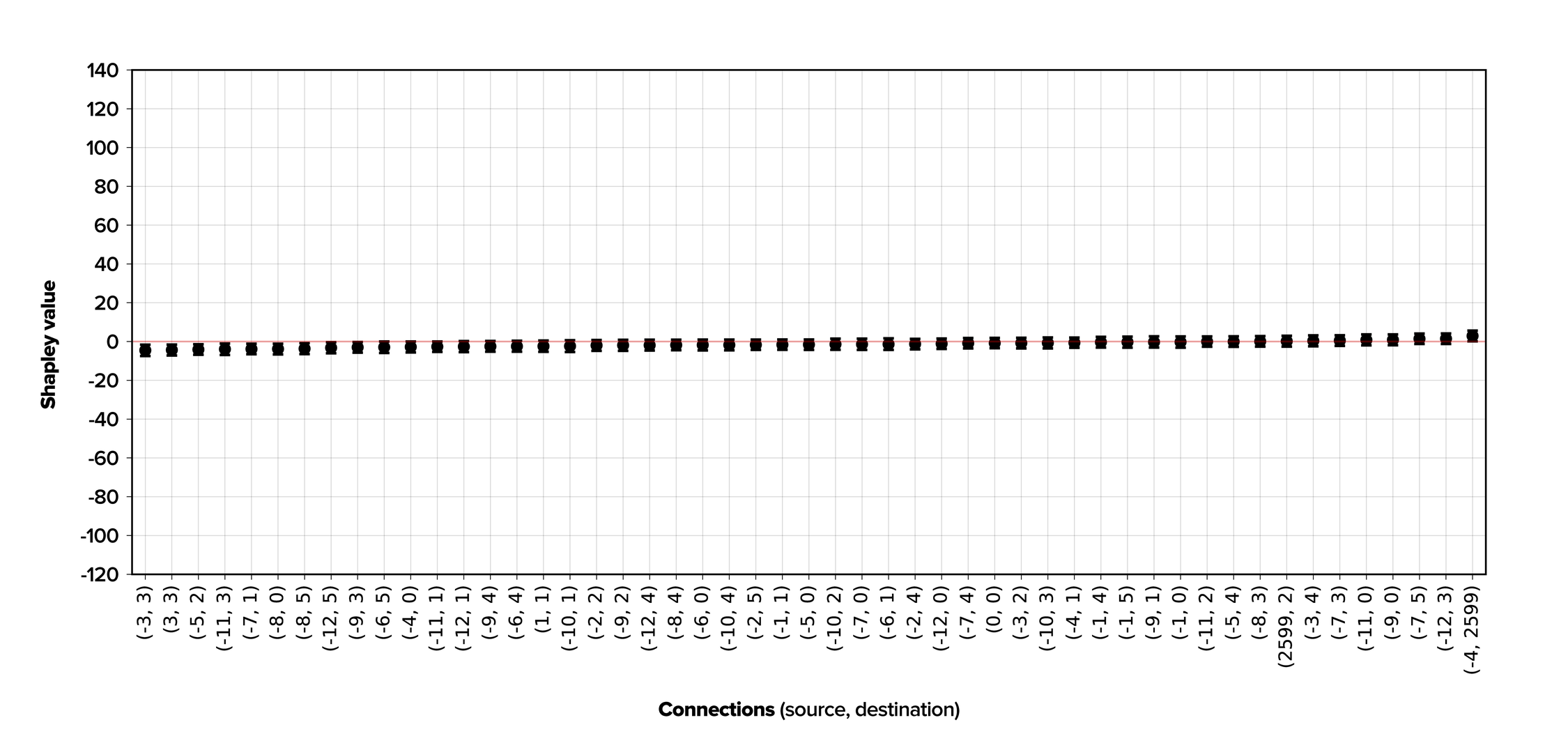

Supplement: S2 Fig — As a sanity check, we performed the MSA on the optimized network connections while feeding it noise instead of game-states. The procedure is explained in the section: Multi-perturbation Shapley value Analysis. We found no connection with considerable causal importance since the network cannot perform properly. (TIF) [file pcbi.1010250.s002.tif]
